# Resting Energy Expenditure of Physically Active Boys in Southeastern Poland—The Accuracy and Validity of Predictive Equations

**DOI:** 10.3390/metabo10120493

**Published:** 2020-12-01

**Authors:** Edyta Łuszczki, Aneta Sokal, Sara Jarmakiewicz-Czaja, Anna Bartosiewicz, Katarzyna Dereń, Maciej Kuchciak, Paweł Jagielski, Artur Mazur

**Affiliations:** 1 Institute of Health Sciences, Medical College of Rzeszów University, 35-959 Rzeszów, Poland; asokal@ur.edu.pl (A.S.); sjczaja@ur.edu.pl (S.J.-C.); abartosiewicz@ur.edu.pl (A.B.); kderen@ur.edu.pl (K.D.); 2 Institute of Physical Culture Sciences, Medical College of Rzeszów University, 35-959 Rzeszów, Poland; mkuchciak@ur.edu.pl; 3 Department of Nutrition and Drug Research, Institute of Public Health, Faculty of Health Sciences, Jagiellonian University Medical College, 31-531 Krakow, Poland; paweljan.jagielski@uj.edu.pl; 4 Institute of Medical Sciences, Medical College of Rzeszów University, 35-959 Rzeszów, Poland; drmazur@poczta.onet.pl

**Keywords:** energy metabolism, exercise, indirect calorimetry, physical activity, resting energy expenditure

## Abstract

Optimization of energy intake in the diet of young athletes is of primary importance. In addition to the energy expenditure associated with their body development, the demand resulting from intensive physical activity also increases. The aim of this study was to compare the accuracy of formulas commonly used for resting energy expenditure (REE) calculations with values obtained from measurements using indirect calorimetry among male children and adolescents practicing football. The study was conducted among 184 boys aged 9 to 17 using a calorimeter and a device for assessing body composition by means of electrical bioimpedance using a segment analyzer. The mean error ranged from −477 kcal/d by the Maffeis formula to −182 kcal/d for the Institute of Medicine of the National Academies (IMNA) formula. A statistically significant difference was found for all formulas in the calculated value in relation to the measured REE value (*p* < 0.0001). Most “ready-to-use” formulas underestimate REE, which can be a risk in determining the total energy demand in a group that requires more calories, especially when due to intensive growth and development and the expenditure associated with regular training and increased physical activity.

## 1. Introduction

Physical activity (PA) plays a significant role in the health and proper development of children, adolescents, and adults [1]. In 2008, a compendium of energy expenditure values among young people was developed [2]. It provides values of metabolic equivalents (MET) for each activity, averaging it out for age, sex and other characteristics and in some cases using adult values [3,4]. However, both physical and physiological features in children differ significantly from adults and even from teenagers. Resting energy expenditure (REE) in children is higher per kilogram of body weight than in adolescents [5].

Optimized energy intake in the diet is crucial for young athletes. In addition, energy expenditure associated with body development and intensive physical activity also increases the demand. Measurement or estimation of REE is usually the first step in determining energy demand, both in the overall population and for people training in various sports disciplines [6]. REE becomes a significant contribution to total energy expenditure even for very active people, such as those training for endurance sports (marathon, swimming, rowing, etc.). For most people, REE accounts for almost 60–70% of total energy demand [7]. Therefore, defining REE can serve as a valuable tool in developing food rations or nutrition plans to improve athletic performance and prevent weight loss in physically active children. Observations made by many authors confirm that the REE is higher in people with greater body weight, who are either overweight or obese [8,9]. Age is also a parameter that has been confirmed in numerous studies to have an influence on the REE [10]. In addition, the strong relationship between the REE and muscle mass has been the subject of research by many authors. The literature shows the FFM as the strongest indicator affecting the REE [11,12]. Adipose tissue is another component of the body composition, and its effect on the REE is also widely analyzed. Some researchers attribute it an important role in influencing energy expenditure [13], while other studies do not show such a relationship [14].

Although REE may be very accurately calculated using laboratory methods such as indirect calorimetry (IC), popular literature formulas are usually used for children and adolescents also in sports. It results from the fact that IC requires expensive professional equipment and is a time-consuming examination. Formulas based on body weight (BW) have been used for over a hundred years [15]. Currently, a broad range of them is available and widely used [16]. However, due to obvious differences in metabolic activity of fat mass (FM) and fat-free mass (FFM), most formulas are based on BW and not on body composition [17]. In athletes, due to the fact that FFM constitutes a larger percentage of body composition, some authors are in favor of using equations based on FFM [18]. It has been shown that FFM can have a strong influence on energy demand. Web [19], in his study, observed a strong correlation between REE and FFM in both women and men. Cunningham et al. [20] found that FFM accounts for about 70% of the variation in REE determination, and FFM-based equations are much more accurate in some populations. However, research on this issue is still inconclusive [21]. Several studies have examined the validity of formulas based on BW or FFM among athletes. Some of them have developed new formulas for specific disciplines. Endurance athletes [22], and a mixed group of athletes from disciplines such as water polo, karate and judo [23], and various team sports such as football, baseball, and swimming [24] were examined. Some of these formulas were developed in well-defined groups of athletes, while others included significant heterogeneity in attempting to create a general formula. It is believed that the accuracy of REE predictive (pREE) formulas is specific to the population for which they were created. Therefore, it indicates that they should not be used for groups other than those originally intended [25]. In addition, despite the convenience and speed of using formulas, they can mask real differences between specific populations of athletes who may differ in body composition or the number of training units affecting REE. Since the vast majority of players are men in football, we decided to examine a group of male children and youth in our study.

The Harris Benedict formula, developed in 1919, is most often used for healthy people. It is often considered as one of the most accurate formulas defining basic energy demand [15]. However, data also indicate that a similar value of pREE to mREE can be obtained using the Mifflin formula. The formula was based on data obtained from a survey of 498 people. The trial covered a wide range of body weight and height. In addition, the subjects were divided according to sex, age, body weight (normal/obesity). Research shows that its use in obese people minimizes the risk of overestimating the demand in this population [26]. In 1986–1987 the Owen formula was also created; however, the sample examined included only adults. Nevertheless, there were also athletes among them [27,28] similarly as in the case of the De Lorenzo formula (1999) [23,29].

The FAO/WHO/UNU formula is most often recommended for children. In 1985, the World Health Organization, Food and Agriculture Organization of the United Nations and United Nations University issued a report in which they present formulas that help in estimating basal energy expenditure, and thus energy and protein requirements. The report presents formulas for adults, newborns, children, adolescents and pregnant and lactating women; thus, the scope of the studied sample was very wide [30]. The basis for the FAO/WHO/UNU formulas was the Schofield database covering 114 studies, approximately 7000 people from 23 countries. Therefore, the results obtained in the analysis may have been influenced by factors such as environment temperature or nutritional status [31]. Another formula that can be used in children was developed by the Institute of Medicine of the National Academies (INNA) in 2002. Data for each age group was used to formulate this, and pREE analysis was based on the doubly labeled water method (DLW) [32]. Molnar’s is another formula that was developed with the participation of children. The examined sample included children of an age similar to this study [33].

The formulas by Altman and Dittmer (1986) [34] and Maffeis (1993) were also based on children’s populations [35]. Some authors in their research indicated that both the Harris-Benedict formula and Altman and Dittmer formula might not be accurate for children with increased adipose tissue because they were developed prior to the development of the “obesity epidemic” and may not include the less metabolic activity of adipose tissue [20,36].

However, they were not developed for physically active children or young athletes. Due to differences in the levels of physical activity and body composition, ready-to-use formulas may not be appropriate for younger athletes. Research to date has shown that formulas can generate errors so large that they affect the final result when estimating energy demand. The literature indicates that a comparison of selected formulas in physically active people with calorimetric testing to confirm their accuracy shows that several of these equations underestimate or overestimate REE by up to 300 kcal [22].

There is still no consensus regarding the appropriate formula used in pediatric populations, especially taking into account factors such as high physical activity or body composition [37,38,39,40]. It is well known that both physical and metabolic status differs from adults in this population, so it is necessary to know the best way to calculate REE in childhood, which, due to its specificity, the dynamic development of children requires meeting not only energy needs but also protein, fats and carbohydrates. To our knowledge, no previous study has examined the accuracy of REE formulas for young footballers. Therefore, the aim of this study was to compare the accuracy of commonly used formulas for calculating REE available in the literature with values obtained from measurement using indirect calorimetry (mREE) among male children and adolescents practicing football.

## 2. Results

### 2.1. Characteristics of the Study Group

A total of 184 boys aged 9 to 17 participated in the study. The mean age of the respondents was 13.20 ± 2.16 years. The results of the measurements taken and an overview of the typical in-season weekly training schedule are presented in Table 1.

### 2.2. The Findings

Table 2 shows the results for the REE calculated by indirect calorimetry and REE measured on the basis of commonly used formulas. The mean REE measured by IC was 1844 kcal, while the mean REE calculated by predicted equations were from 1368 kcal (Maffeis formula) to 1662 kcal (IMNA formula). All equations underestimated the REE. The mean error ranged from −477 kcal/d for the Maffeis formula to −182 kcal/d for the IMNA formula. The IMNA equation gave the most accurate REE predictions in physically active boys, with more than 50% accurate predictions and CCC 0.72.

The Cunningham equation, which was the only formula based on FFM (Table 4), underestimated the REE in −395 kcal/d with an accuracy of 7.6%.

A statistically significant difference was found for all formulas in the calculated value in relation to the measured REE value (*p* < 0.0001).

**Figure 1:**
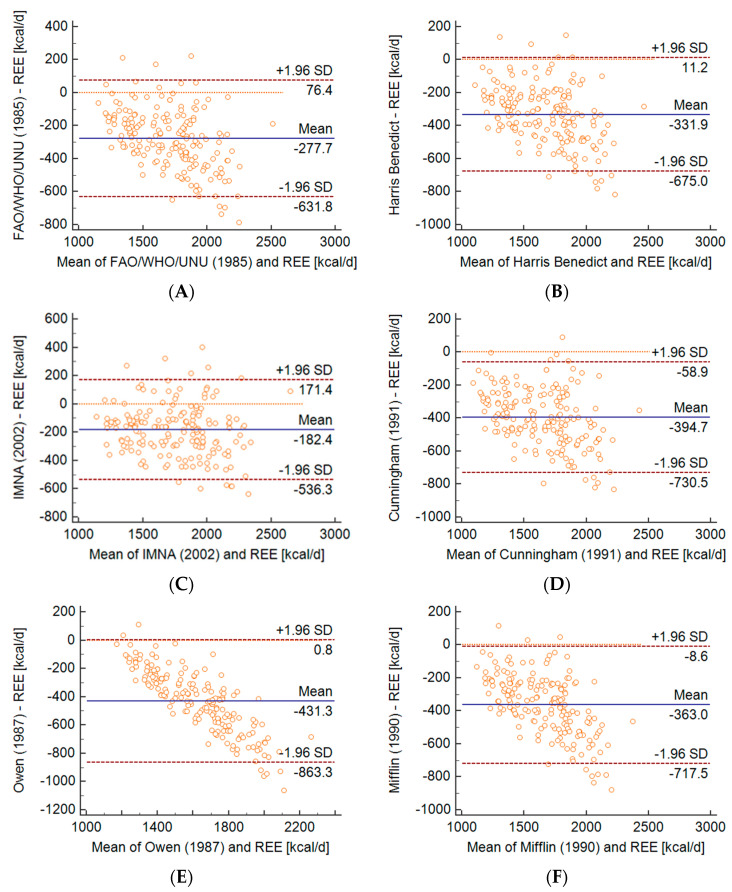
Bland-Altman plots presenting the agreement between measured and predicted resting energy expenditure (REE) by the equations of (**A**) FAO/WHO/UNU, (**B**) Harris Benedict, (**C**) IMNA (Institute of Medicine of the National Academies), (**D**) Cunningham, (**E**) Owen, (**F**) Mifflin. All of the formulas above were used in the selection of children who regularly exercise. The mean value of the differences between measured and predicted REE (bias) is indicated by the solid line. The dashed lines delimit the 95% confidence interval. All regression lines were statistically significant at *p* < 0.0001, indicating a systematic bias.

**Figure 2:**
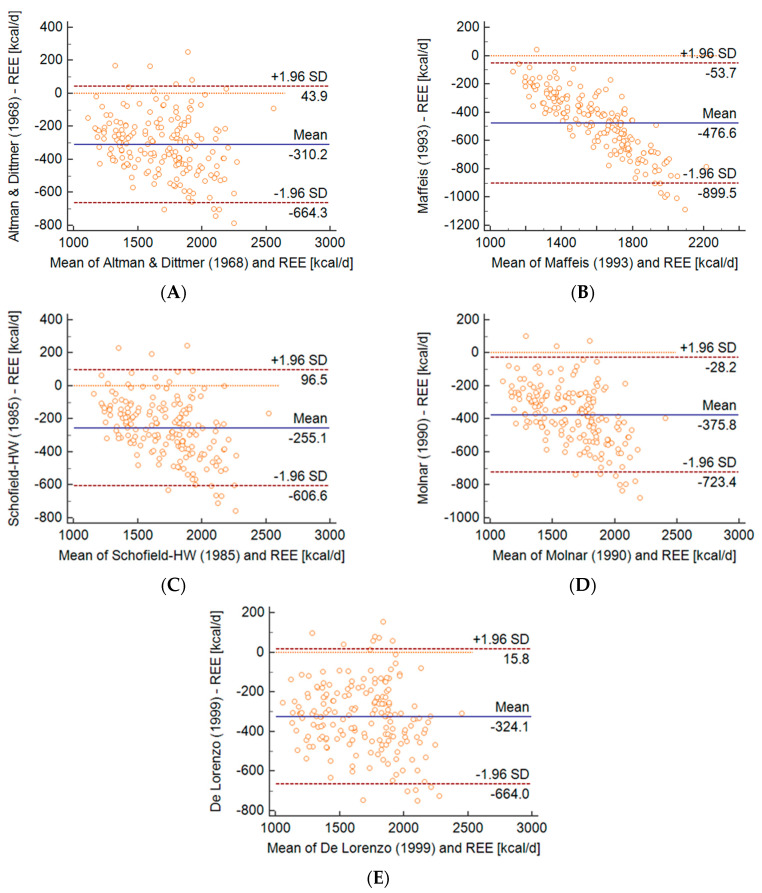
Bland-Altman plots the presenting the agreement between measured and predicted REE by the equations of (**A**) Altman and Dittmer, (**B**) Maffeis, (**C**) Schofield Henry William (Schofield-HW), (**D**) Molnar, (**E**) De Lorenzo. All of the formulas above were used in the selection of children who regularly exercise. The mean value of the differences between measured and predicted REE (bias) is indicated by the solid line. The dashed lines delimit the 95% confidence interval. All regression lines were statistically significant at *p* < 0.0001, indicating a systematic bias.

The Bland–Altman analysis (Figure 1 and Figure 2) was used to assess the compliance of the results from REE measurements using the IC and prediction formulas. The IMNA equation underestimated the results to the least extent (50% of the respondents) with a significant deviation of 9.5%. On the other hand, the values obtained using the Maffeis formula gave the largest error (3.8% of respondents) with a significant deviation of 19.8% (Figure 1C and Figure 2B). However, the 9 formulas used in this study were also underestimated (13.2–22.1%) (Figure 1 and Figure 2).

## 3. Discussion

To the best of our knowledge, there are very few studies available in the literature regarding the assessment of REE using indirect calorimetry in healthy children practicing sport regularly. There is also very little research on the accuracy of using ready-made formulas for calculating REE in this group. As demonstrated by our results, the measured REE using indirect calorimetry significantly differed in the study group from the basal metabolic rate calculated using commonly used formulas available in the literature. All the formulas we used in the study underestimated the value of resting metabolism. From a practical perspective, it is hoped that these data will be a guide to other researchers that when it is possible, the measurement of REE is always recommended.

Our study group consisted of 184 young males. This was due to the fact that the school that was selected for the study had a football program that is currently a more popular sport among men. The mean REE value for physically active boys was 1844 kcal/day in this study, which seems to be representative of other active men tested so far. In previous studies among active men, obtained values were 1858 kcal/day, 1788 kcal/day and 1808 kcal/day [41,42,43]. In the study of resting energy expenditure among 10 footballers, the results of REE was 1834 kcal/day [36]. In addition, the average REE in 24 endurance athletes was around 1868 kcal/day [22]. Out of 11 prognostic formulas evaluated in this study, all formulas, except for the Cunningham formula, included parameters such as body height, weight and age as variables. Based on the results of our study, the formula that could most accurately predict REE values in the study group of young men was the IMNA formula producing the lowest error value (−182 kcal; −9.5%). This value is a limit value not exceeding 10% of the error; therefore, the IMNA formula seems to be the right one to be used in young players in our group. Our results show that this formula functions well in male children and is consistent with the study of Jae-Hee et al. Nevertheless., in their study, this formula was better suited to physically inactive children [44]. Although the IMNA equation was developed to estimate REE in overweight people [32], the fact that it was appropriate in our study may be due to the fact that REE correlates closely with body weight and that its higher level can be seen in a person with higher body weight [45]. However, in the case of people who regularly engage in intense aerobic exercise, the post-exercise REE may increase [46].

In the case of the Cunningham formula, the mean error was as much as −395 kcal despite the fact that in the papers of other researchers, this formula reached lower error ranges within the 160 kcal limit. However, it was mainly used by the authors on a group of adult athletes [22,24]. Thus, it turned out in our study that the FFM-based formula was not the best predictor of REE in the group of young athletes.

The error in our study was similar in the case of the Harris Benedict formula. It underestimated REE values by 332 kcal on average (bias −17.5%). In most studies, this formula is a very good predictor of determining REE since it was based on a young population of women and men with normal body weight [8]. This prognostic model includes many elements, such as height, weight and age, all of which affect REE [47]. Hence it proved to be accurate in many studies, also in the group of athletes, although the group consisted of adults [18].

The literature indicates that formulas having the smallest error deviations in the population of children and adolescents are the formulas of Schofield and FAO/WHO/UNU based on body weight and height. Rodriguez et al. presented that the FAO/WHO/UNU and Schofield formulas were suitable for predicting REE in children and adolescents [48]. A study by Hofsteenge et al. showed that it is not a good equation for obese adolescents and showed overestimation with a deviation of + 10.7% and mean squared prediction error 276 kcal/d [49].

On the other hand, a meta-analysis from 2020 among obese children and adolescents showed that the Mifflin formula shows the highest precision in the 11–18 age group [50]. In our study, the Schofield and FAO/WHO/UNU formulas reached similar error values (bias −13.2% and −14.4%, respectively), while the Mifflin formula as much as −19.0% (−363 kcal/d).

We can assume that the body composition profile of our participants could differ from other children and adolescents of this age. This may be the reason why the formulas most commonly used in children (e.g., FAO/WHO/UNU) are not appropriate in our study group. It is presumed that the individual components of body weight, both tissues and organs, may have different metabolic rates [51,52]. Therefore, most of the interindividual variability in REE can be accounted for by differences in FFM [13]. Nevertheless, specialized tests are still needed to directly assess the metabolic rate of individual body components [53]. Along with age and gender, physical activity has a major influence on the amount and proportion of FFM. The inclusion criteria of our study were to train about 3 times per day and playing a match once a week. Therefore, about 10.5 h/week physical activity were calculated. This is more than the average physical activity of non-athletic children and adolescents [54]. However, limited evidence exists about the evaluation of body composition and its dynamic changes in children practicing sports [55]. In addition, poor data exist in the literature about the comparison of the body composition between healthy non-athletic and athletic young individuals by means of BIA evaluation [56,57,58]. Therefore, future studies are needed to evaluate the impact of training and physical activity on body composition in athletic children and adolescents.

There are many challenges involved in determining the accuracy of individual formulas in the population of children and adolescents. They include dynamic changes in the body, rapid growth, differentiation of body composition, as well as the effect of puberty on REE during adolescence [59]. The growth spurt of men comes relatively late compared to girls. It is characterized by a greater linear growth and deposition of lean mass. Many cross-sectional and longitudinal studies have shown that REE and total daily expenditure grow during puberty. On the other hand, the scientific evidence based on FFM-adjusted energy values is limited, and the main emphasis here is on the role of the rise in the level of hormones: insulin-like factor-1 (IGF-1), testosterone, growth hormone (GH) and changes in thyroid function [60].

In summary, the results of the current study indicate that the majority of the REE prediction equations being examined underestimated resting energy expenditure with the exception of the IMNA [32] equation in youth athletes. Therefore, based on the REE prognostic formulas for energy demand recommendations, it is valuable to remember that they may underestimate the energy demand of a young athlete. Similar results were found in other studies checking the accuracy of REE prognostic formulas in sporting populations, especially in endurance sports [18,22,23,24]. For example, De Lorenzo et al. [18] found that the Cunningham equation overestimates (+59 kcal/d) measured REE. Harris Benedict and Mifflin formulas underestimated REE in 51 male athletes in various types of sports. Likewise, Thompson et al. [22] presented that all average predicted REE values were lower than those measured with indirect calorimetry, except for the Cunningham formula in male and female endurance athletes [24].

The reason why predictive formulas for REE underestimate real REE is not entirely clear; however, there are several factors that can affect this state. Athletes are more physically active due to regular training sessions and matches, which may affect elevated REE values compared to people who are less active or have a sedentary lifestyle, as it has been shown that increased physical activity, especially when taking place recently, affects REE values [61,62,63]. The literature indicates that recent physical activity can affect REE even 72–96 h after exercise [32,47,64], which may also explain why some prognostic formulas for REE underestimate realistically measured REE using indirect calorimetry. It also highlights the need for more specific formulas to calculate REE in various sports to better reflect fluctuations in metabolic activity after physical activity. In addition, an increase in physical activity increases FFM, which has been presented to account for a large proportion of the measured REE variability and is positively correlated with REE [20,22,23,24].

There are also a number of potential limitations of the study that must be taken into account when interpreting the results. First, this was a cross-sectional study, so temporality and causality issues could not be considered. The selection error could be due to the fact that only one sports school was included in the study. In the future, the study should be extended to more schools. The results are the basis for determining REE only in a specific population in this one sport (football), male, Caucasian and at a specific age. Therefore, the current findings could not be generalized. Then, we did not assess eating habits in the days prior to the REE measurement, although this may influence the RQ of the patient. Additionally, the total energy expenditure is rarely adjusted according to individual needs; the actual calorie deficit per individual is an important confounding variable. The group consisted of youth athletes meaning our results could be confounded by overweight/obesity and unintentional weight gain. While participants were instructed to exercise their school sports program, we could not verify adherence to this instruction, which precludes determining whether changes in physical activity patterns affected study outcomes. Epidemiological and laboratory studies have consistently found that sleep loss can change energy expenditure by affecting each of its components: resting energy expenditure, diet-induced thermogenesis and physical activity. Poor sleep quality and its deficit may result in higher energy and fat intake, which was closely related to obesity development and increased REE in some studies [65,66]. In future studies, the impact of sleep duration on resting energy expenditure should be investigated. Another limitation of the study is the use of the electrical impedance method to assess body composition. Although it is used in the literature on a large scale, the method of assessing body composition using densitometry would allow for even more precise results. In our study, a portable device (Fitmate Med) that allows for easier measurement of REE and with a lower cost was employed. Fitmate Med using a constant RQ may introduce differences in the estimate of oxygen consumption and REE values. Compared to the Douglas bag method, the literature demonstrated no significant differences in oxygen consumption. On this basis, it can be assumed that in our study, the Fitmate Med device may slightly overestimate the REE value, but this difference is within the limits of the statistical error. Despite not measuring CO_2_ production, it is very convenient in the clinical setting, assuming a minimal error of analysis. Finally, we measured each subject only once; thereby, we could not estimate the intra-and inter-individual variation in REE.

## 4. Materials and Methods

### 4.1. Subjects

**Figure 3:**
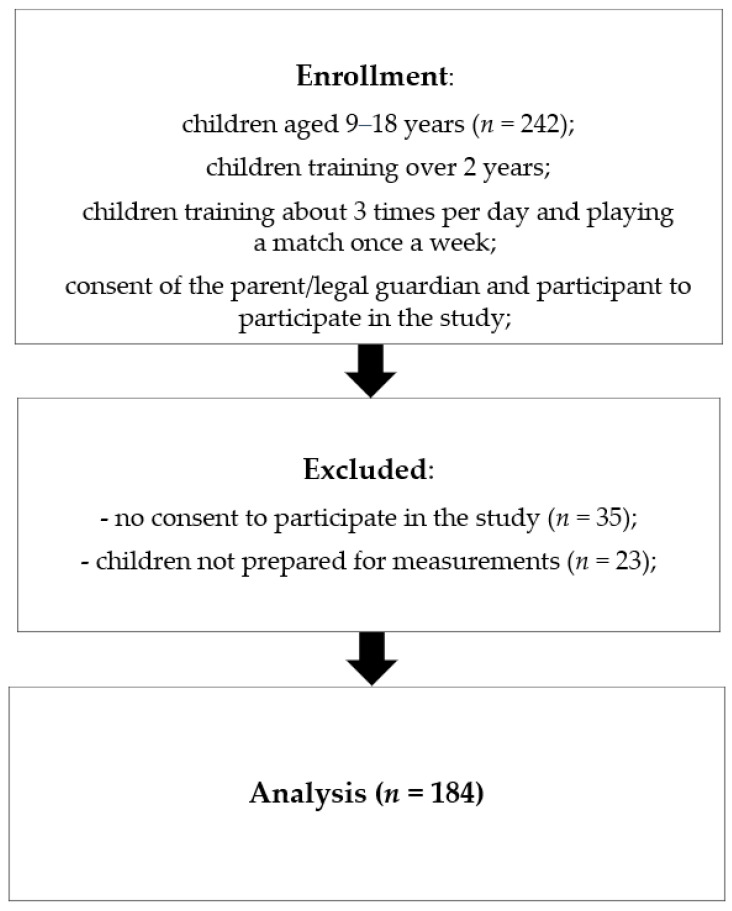
Selection of the study participants.

The study involved healthy children aged 9–17 from a sports school (Sports Championship School, Rzeszów, Poland), located in south-eastern Poland during the 2018/2019 school year. In order to select the school, invitations were sent to all (7 schools) of the sports schools in the area (Podkarpackie Voivodeship, Poland). Out of those that agreed (3 schools), one school was selected using a randomized algorithm. The sample size was determined with the help of the EPI INFO (StatCalc) software. Assuming there were 2000 pupils in the sports schools in Podkarpackie Voivodeship, we estimated that the sample should include 179 children, with a confidence level of 95% and 7% margin of error. The study was conducted in a randomly selected school. A multistage random cluster sampling method was used. Invitations to participate in the study were sent to 242 parents or guardians of boys attending this school. The inclusion criteria were as follows: male, practicing football, training regularly, aged 9–18, and with the consent of the parent/guardians to participate in the study. The selection of study participants is presented in Figure 3. All subjects were healthy, without a history of chronic diseases, no weight loss in the last 6 months, no feverish diseases over the last month. They also did not use any pharmacological or hormonal treatment. All participants and their legal guardians received verbal and written information about the objectives, risks and profits of the study. Both guardians and participants gave their informed consent to participate in the study. Two hundred seven consents were received from study participants. Of these responses, 23 participants were excluded from the study because they were not prepared for measurement. Finally, the study group consisted of 184 boys aged 9–17 years.

### 4.2. General Procedures

The tests were carried out at a controlled temperature (22–25 °C) in the Laboratory of Innovative Research in Dietetics (Center for Innovative Research in Medical and Natural Sciences, University of Rzeszów, Rzeszów, Poland). All measurements were performed by experienced researchers. Participants reported to the laboratory in the morning between 6:00 and 8:30.

#### 4.2.1. Anthropometric Measurements, Body Composition and Body Mass Index

The first stage of the study included anthropometric measurements and body composition analysis. At the beginning, the subjects were informed of the entire test procedure, including the need to empty the bladder as required to minimize the risk of error during body composition analysis. Initially, height measurement was made with a height meter (Seca 213 portable stadiometer) with an accuracy of 0.5 cm. For measurement, the subjects were asked to remove their footwear and stand with their back to the stadiometer in an upright position. The average of three measurements was used for analysis. The analysis was performed by means of the electric bioimpedance method (6.25 kHz, 50 kHz, 90 µA) using a calibrated segment analyzer (Tanita MC-980 PLUS MA, Tokyo, Japan) with an accuracy of 0.1 kg/0.1%. The results obtained using the Tanita analyzer for studies involving children are consistent with those obtained from dual-energy X-ray absorptiometry (DXA) [67,68,69,70]. The analyzer is equipped with 8 electrodes, of which 4 are built into the platform, while the others are placed in the handles. Subjects were asked to remove footwear and socks. Next, the skin on their soles was cleaned so that the measurement was carried out correctly. Measurements were made in underwear, standing motionless in designated places on the platform. According to the Tanita MC-980 PLUS MA manual, accurate measurement requires setting up the machine as level as possible. The adjustable feet were rotated in 4 positions so that the bubble of the level indicator was in the middle. Participants stood on the platform barefoot, upright, legs extended, placing their feet so that they touched the front and rear electrodes, making sure that the weight of the body is evenly distributed over both feet. The examined person held handles in their hands abducted from the body at an angle of 35°–40°.

Body mass index (BMI) was calculated as body weight (kg)/height (m)^2^. Definitions of body mass deficiency, normal body weight, overweight and obesity were based on the Centers for Disease Control and Prevention recommendations [71].

#### 4.2.2. Resting Energy Expenditure Assessment by Indirect Calorimetry

REE was measured by indirect calorimetry using a portable calorimeter (Fitmate MED, Cosmed, Rome, Italy). The device was calibrated according to the instructions provided by the manufacturer using a 3-L syringe (Cosmed; P/N: C00600-01-11). A turbine flowmeter was used to measure ventilation, while the analysis of exhaled oxygen was possible due to the presence of a galvanic cell sensor. VO_2_ calculations were based on the so-called Haldane principle through the formula: IV = (100 + R ×(FeO_2_ − 20.93) − FeO_2_) ×EV/79.04 (where: IV = inspiratory volume, EV = expiratory volume, FiO_2_ = fraction of inhaled O_2_, FeO_2_ = average O_2_ concentration in exhaled air). The ratio of CO_2_ produced to O_2_ determines the respiratory quotient (RQ). The respiratory ratio (R) during the study was 0.85. The RQ value comes from averaging the values resulting from the oxidation of energy substrates, which are 1.0 for the protein and 0.8 and 0.7 for the fat diet [72]. VCO_2_ is not measured directly; it is estimated with a constant respiratory rate (RQ) of 0.85. Fitmate monitors oxygen uptake (VO_2_), the fraction of O_2_ expired (FeO_2_), ventilation (Ve), heart rate (HR) and respiration rate (Rf). Estimation of REE (kcal/day) is possible thanks to the modified Weir equation: REE = [5.675 × VO_2_ + 1.593 × VCO_2_ − 21.7]/; VO_2_ is the oxygen volume in the breath (mL/min), and VCO_2_ is the carbon dioxide output (mL/min) [73].

The test was carried out at room temperature in a ventilated room and quiet environment. Before the measurement, the participants were presented with the entire test procedure; in addition, each participant lay on his back for 30 min to acclimatize to the environment. REE was measured using a medical-grade cart (Cosmed, Rome, Italy) and suitable silicone rubber pediatric masks (Cosmed, Rome, Italy). To assess REE, patients were placed supine on a couch (Norma II, Juventus, Poland) with a pillow placed under the head. Participants were instructed to lie still, avoid speaking and not fall asleep during measurement. The Fitmate Med device was validated [74] and showed very high reliability of measurements obtained [75]. The results obtained using Fitmate Med are comparable to those obtained by the Douglas bag system, which uses a sensor to measure VCO_2_ [74].

According to the instructions, it was recommended to use disposable antibacterial filters with rubber mouthpieces to improve the mouth grip and limit the risk of air leaking by a reusable mask (a petite/pediatric size).

The evidence-based protocol for measurement of resting energy expenditure by indirect calorimetry [76] was adopted in the study and clearly discussed (Table 3).

#### 4.2.3. Predictive REE Equation

To estimate REE, commonly used formulas of various forms were used with data such as body weight, height, or age, but some of them included FFM (Cunningham formula), which was also used for the following analysis. When choosing the formulas, different populations (ethnicity, climate, body structure) at different periods of time, which is important, i.e., from the point of view of increasing overweight and obesity, were taken into account. Equations evaluated in the study are presented in Table 4.

### 4.3. Statistical Analysis

The study results were obtained using descriptive statistics: number (n), Me—median, and standard deviation (SD). The choice of the parametric test depended on fulfilling its basic assumptions, i.e., the conformity of the tested variable with normal distribution, which was verified by the Shapiro–Wilk test. Because of normally distributed variables, differences between REE and theoretical values calculated from individual formulas were checked using the t-test for dependent samples. Bias was calculated as the mean difference between the predicted value and the measured REE. The agreement between measured and estimated REE values was evaluated by determining the bias in absolute values and as a percentage of the measured value and the corresponding limit of agreement (upper limit of agreement (ULA) = bias + 1.96 × SD; lower limit of agreement (LLA) = bias − 1.96 × SD). Additionally, Pearson’s product-moment correlation coefficient (r) and the coefficient of determination (R^2^) were calculated. The concordance correlation coefficient (CCC) as a measure of agreement was also determined. The percentage of participants whose predicted REE value fell within ±10% of the measured REE was taken as a measure of accuracy. The heteroscedasticity was tested using the Bland–Altman method [77]. The plots presented the difference between predicted and measured REE versus the mean of predicted and measured REE. The statistical analyses were performed using PS MAGO PRO 6.0 (IBM SPSS STATISTICS 25) and MedCalc. Statistical significance was set at *p* < 0.05.

### 4.4. Ethics

This research project was carried out in accordance with the Helsinki Declaration. The study was approved by the institutional Bioethics Committee at the University of Rzeszów (Resolution No. 2/01/2019) and by all appropriate administrative bodies.

## 5. Conclusions

In conclusion, the best predictive equation for children who engage in regular physical activity has not been created so far. Although the use of the IMNA formula gave the smallest error in the REE estimates, this formula and all of the predictive equations used in this study underestimated the energy needs of children. Therefore, the more convenient way to assess REE in this group is to measure it using the indirect calorimetry method. Due to the still low availability of IC devices, there is a need for further research that will allow the creation of a special formula for regularly physically active children.

## Figures and Tables

**Table 1:** Characteristics of the study group.

Variables	Mean	SD	Me	Min	Max
REE (kcal)	1844.42	327.97	1883.00	1183.00	2639.00
Age (years)	13.20	2.16	13.00	10.00	16.00
Height (cm)	162.91	14.90	166.00	132.00	191.00
Weight (kg)	52.37	14.44	53.85	24.80	102.20
BMI (kg/m^2^)	19.27	2.57	19.05	13.60	28.00
Fat (%)	17.58	3.76	16.90	8.80	34.00
Fat (kg)	9.20	3.29	8.60	4.00	22.60
FFM (kg)	43.17	12.02	44.40	20.60	79.60
TBW (kg)	31.84	9.10	32.75	15.10	58.80
PA (h/week) ^a^	10.69	0.59	10.50	10.00	12.00

^a^ Physical activity includes pitch-based training (technical and tactical soccer-specific training, with volume and intensities specific to the age group’s competitive soccer demands ~90 min/4 times per week); gym-based training (movement competency exercises ~45 min/4 times per week); competitive match-play (90 min/once per week). Me—median; SD—standard deviation; Min—sample minimum; Max—sample maximum. REE—resting metabolic rate; BMI—body mass index; FFM—fat-free mass; TBW—total body water; PA—physical activity.

**Table 2:** Validity of resting energy expenditure.

Equation	REE (kcal/d)		*t*-Test	Bias kcal/d		LLA kcal/d	ULA kcal/d	Bias (%)		r	*p*-Value (Correlation)	R^2^	*p*-Value (Linear Regression)	CCC	Prediction		
	Mean	SD	*p*-Value	Mean	SD			Mean	SD						Accurate%	Under%	Over %
REE	1844	328													100		
Harris Benedict	1513	256	<0.0001	−332	175	−675	11	−17.5	8.0	0.84	<0.0001	0.72	<0.0001	0.50	14.7	84.8	0.5
FAO/WHO/UNU (1985)	1567	250	<0.0001	−278	181	−632	76	−14.4	8.4	0.84	<0.0001	0.70	<0.0001	0.56	25.0	73.4	1.6
IMNA (2002)	1662	303	<0.0001	−182	181	−536	171	−9.5	9.3	0.84	<0.0001	0.70	<0.0001	0.72	50.0	46.7	3.3
Cunningham (1991)	1450	264	<0.0001	−395	171	−731	−59	−21.1	7.6	0.85	<0.0001	0.73	<0.0001	0.44	7.6	92.4	0.0
Mifflin (1990)	1481	224	<0.0001	−363	181	−717	−9	−19.0	7.6	0.84	<0.0001	0.72	<0.0001	0.43	9.2	90.8	0.0
Owen (1987)	1413	147	<0.0001	−431	220	−863	1	−22.1	8.6	0.84	<0.0001	0.70	<0.0001	0.26	8.7	91.3	0.0
Altman and Dittmer (1968)	1534	283	<0.0001	−310	181	−664	44	−16.5	8.8	0.84	<0.0001	0.70	<0.0001	0.55	19.0	79.3	1.7
Maffeis (1993)	1368	150	<0.0001	−477	216	−900	−54	−24.7	7.8	0.85	<0.0001	0.72	<0.0001	0.23	3.8	96.2	0.0
Schofield-HW (1985)	1589	253	<0.0001	−255	179	−607	96	−13.2	8.5	0.84	<0.0001	0.71	<0.0001	0.59	30.4	67.9	1.7
Molnar (1990)	1469	239	<0.0001	−324	173	−723	−28	−19.8	7.6	0.85	<0.0001	0.72	<0.0001	0.43	10.3	89.7	0.0
De Lorenzo (1999)	1520	298	<0.0001	−376	177	−664	16	−17.5	8.7	0.84	<0.0001	0.72	<0.0001	0.55	17.4	82.6	0.0

REE—measured resting energy expenditure by indirect calorimetry; SD—standard deviation; *t*-test—*t*-test for dependent samples; Bias—mean difference between the predicted value and the measured REE; LLA—lower limit of agreement; ULA—upper limit of agreement; Bias%—mean bias in%; r—Pearson’s correlation coefficient; R^2^—coefficient of determination; CCC—concordance correlation coefficient; accurate%—percentage of subjects in which the error of the predictive equation was within 10% of the measured value; Under%—percentage of subjects underestimated by the predictive equation with an error >10% of the measured value; Over%—percentage of subjects overestimated by the predictive equation with an error >10% of the measured value; *p*-value—indicate significant values (*p* < 0.05).

**Table 3:** Evidence-based guidelines for measurement of resting metabolic rate with indirect calorimetry [76].

Criteria	Guidelines for Measurement	Study Group Recommendation
Fasting (thermic effect of food)	Minimum fast 5 h after meals or snacks (grade II) ^a^, 4 h after small meal if longer fast is clinically inappropriate (grade II)	All recommendations concerning preparations for the study were outlined, including having rest for a minimum of 20 min, abstention from nicotine for a minimum 2 h, refraining from the consumption of meals 12 h before the test, refraining from drinking beverages with caffeine and alcohol content for the last 48 h before the test, as well as refraining from participation in physical activity for the previous 14 h. The method of conducting the study was explained in detail, and each study participant had the opportunity to visit the test rooms beforehand and familiarize themselves with the equipment so that it did not raise concerns or cause anxiety in the researched group.
Alcohol ingestion	Minimum abstention from alcohol for 2 h (grade III)	
Nicotine ingestion	Minimum abstention from nicotine for 2 h (grade II)	
Caffeine ingestion	Minimum abstention from caffeine for 4 h (grade II)	
Rest periods	Rest 10–20 min (grade III)	
Physical activity restriction	Minimum abstention from moderate aerobic or anaerobic exercise for 2 h before the test (grade II), for vigorous resistance exercise abstention of at least 14 h (grade III)	
Environmental conditions	Allow a room temperature of 20 °C–25 °C (68° F–77° F) (grade III) Ensure each individual is physically comfortable with measurement position during the test and repeated measures are in the same reclined position (grade V)	The rooms had a controlled temperature between 22 to 25 °C. In addition, each participant had the opportunity to acclimatize to the environment by lying flat for 30 min.
Gas collection devices	Use rigorous adherence to prevent air leaks (grade III). Further studies comparing modern gas collection devices are needed in healthy and clinical populations (grade V)	REE was measured using suitable silicone rubber pediatric masks (Cosmed, Rome, Italy) to ensure maximum sealing and prevent air leakage. This is essential for correct measurement.
Steady-state conditions and measurement interval	Discard initial 5 min. Then achieve a 5 min period with 10% CV ^b^ for VO_2_^c^ and VCO_2_^d^ (grade II)	We use a 10-min protocol in which the first 5 min of data are discarded, and the remaining 5 min of data have a coefficient of variation of no more than 10%.
No. of measures/24 h	Achieve steady state, and one measure is adequate; if not, two to three nonconsecutive measures improve accuracy (grade II)	1 measure/24 h
Repeated measures (daily to monthly variation)	Repeated measures vary 3–5% over 24 h (grade II) and vary up to 10% over weeks to months (grade II)	-
Respiratory quotient (RQ)	RQ measures 0.70 or 1 suggest protocol violations or inaccurate gas measurement (grade II)	The Fitmate employs a turbine flowmeter for measuring ventilation and a galvanic fuel cell oxygen sensor for analyzing the fraction of oxygen in expired gases and uses standard metabolic formulas to calculate oxygen uptake. VCO_2_ is not measured directly but estimated assuming a fixed respiratory quotient (RQ) of 0.85.

^a^ Grade I—strong, consistent evidence; grade II—somewhat weaker evidence and disagreement among authors may exist; grade III—limited design quality; grade IV—professional opinion only, no clinical trials; grade V—no available studies. ^b^ CV—coefficient of variation (standard deviation [mean of individual replicate measures] × 100). ^c^ VO_2_—oxygen consumption. ^d^ VCO_2_—carbon dioxide production.

**Table 4:** Equations evaluated in the study.

Name [kcal/day] Equation for Males	Study Population *
Harris Benedict REE = 66.473 + [13.752 × weight] + [5.003 × height] − [655.093 × age]	136 men, 103 women, 94 infants; normal body mass [15]
FAO/WHO/UNU REE = 16.6 × weight + (77 × height/100) + 572)	participants, including approx. 7500 children [30]
IMNA REE = 68 − [43.3 × age] + [712 × (height/100) + [19.2 × weight]	1242 participants normal body mass; for people with moderate physical activity; a diverse group of respondents [32]
Cunningham REE = (22 × fat-free mass) + 500	120 men, 103 women; normal body mass [20]
Mifflin REE = [9.99 × weight] + [6.25 × height] − [4.92 × age] + 5	251 men, 247 women; a diverse group of respondents including obese individuals [26]
Owen REE = 879 + [10.2 × weight]	60 men, 44 women; a diverse group of respondents including obese individuals [27,28]
Altman and Dittmer REE = [(0.0818 × weight) + 21.09] × 24	>200–300 children aged 3–16 years old, average weight for height [34]
Maffeis REE = [1287 + (28.9 × weight) + (23.6 × height) − (69.1 × age)]/4.18	130 children (62 boys and 68 girls), including 97 normal body mass and 33 obese; 6–10 years [35]
Schofield-HW REE = [16.245 × weight] + [1.371 × height] + 515.3	3575 men, 1239 women; a diverse group of respondents; including about 1000 young male Italian soldiers and cadets [31]
Molnar REE = (12.16 × weight) + (6.04 × height) − (12.02 × age) + 6.43	193 children (116 non-obese and 77 obese); aged 10–16 years [33]
De Lorenzo REE = 857 + [9.0 × weight] + [11.7 × height]	51 men, athletes, practicing sport 3 × a day [27,28]

REE—the value of resting energy expenditure obtained from the predictive formula (kcal/day). * column contains information about the size of the studied group on the basis of which the formula was originally developed.
